# 5-Acetyl-4-(2-chloro­phen­yl)-6-methyl-3,4-dihydro­pyrimidine-2(1*H*)-thione

**DOI:** 10.1107/S1600536809047187

**Published:** 2009-11-14

**Authors:** N. Anuradha, A. Thiruvalluvar, K. Pandiarajan, S. Chitra, R. J. Butcher

**Affiliations:** a PG Research Department of Physics, Rajah Serfoji Government College (Autonomous), Thanjavur 613 005, Tamil Nadu, India; b Department of Chemistry, Annamalai University, Annamalai Nagar 608 002, Tamil Nadu, India; c Department of Chemistry, Howard University, 525 College Street NW, Washington, DC 20059, USA

## Abstract

In the title mol­ecule, C_13_H_13_ClN_2_OS, the heterocyclic ring adopts a flattened boat conformation with the plane through the four coplanar atoms making a dihedral angle of 85.6 (1)° with the benzene ring, which adopts an axial orientation. The thionyl, acetyl and methyl groups all have equatorial orientations. Inter­molecular N—H⋯O, N—H⋯S and C—H⋯S hydrogen bonds are found in the crystal structure. A weak C—H⋯π inter­action involving the benzene ring also occurs.

## Related literature

For chemical and biological applications of dihydro­pyrimidinones, see: Atwal *et al.* (1990 ▶); Kappe (1993 ▶, 2000 ▶); Kappe *et al.* (2000 ▶); Rovnyak *et al.* (1995 ▶); Sadanandam *et al.* (1992 ▶). For related crystal structures, see: Anuradha *et al.* (2008 ▶, 2009 ▶); Chitra *et al.* (2009 ▶).
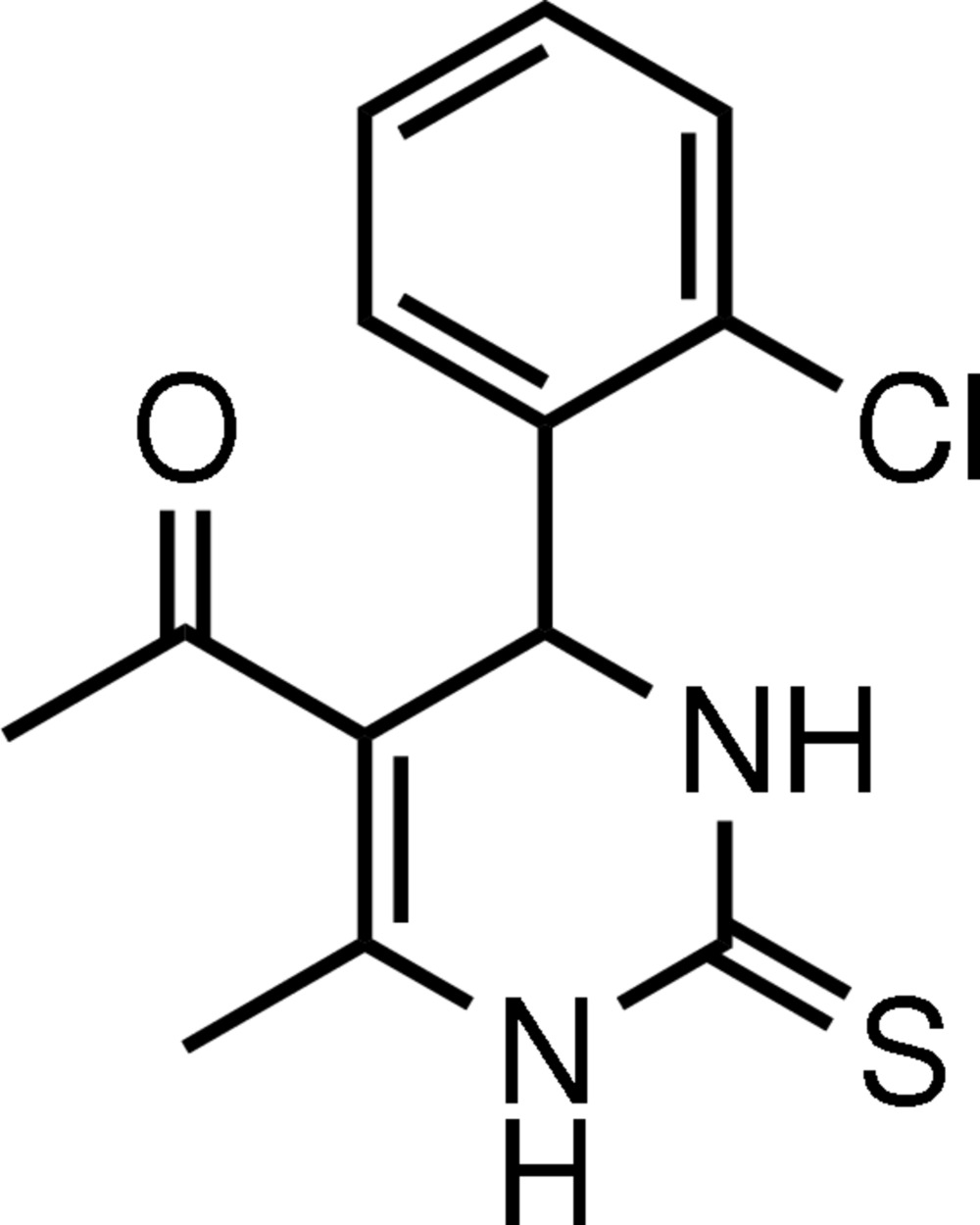


## Experimental

### 

#### Crystal data

C_13_H_13_ClN_2_OS
*M* *_r_* = 280.77
Monoclinic, 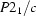

*a* = 7.2346 (12) Å
*b* = 22.585 (3) Å
*c* = 8.0941 (15) Å
β = 106.177 (17)°
*V* = 1270.2 (4) Å^3^
*Z* = 4
Cu *K*α radiation
μ = 4.11 mm^−1^
*T* = 110 K
0.45 × 0.43 × 0.12 mm

#### Data collection

Oxford Diffraction Xcalibur Ruby Gemini diffractometer
Absorption correction: multi-scan (*CrysAlis Pro*; Oxford Diffraction, 2009 ▶) *T*_min_ = 0.451, *T*_max_ = 1.000
4641 measured reflections
2497 independent reflections
2246 reflections with *I* > 2σ(*I*)
*R*_int_ = 0.035

#### Refinement

*R*[*F* ^2^ > 2σ(*F* ^2^)] = 0.062
*wR*(*F* ^2^) = 0.184
*S* = 1.13
2497 reflections
173 parameters
1 restraint
H atoms treated by a mixture of independent and constrained refinement
Δρ_max_ = 1.19 e Å^−3^
Δρ_min_ = −0.39 e Å^−3^

### 

Data collection: *CrysAlis Pro* (Oxford Diffraction, 2009 ▶); cell refinement: *CrysAlis Pro*; data reduction: *CrysAlis Pro*; program(s) used to solve structure: *SHELXS97* (Sheldrick, 2008 ▶); program(s) used to refine structure: *SHELXL97* (Sheldrick, 2008 ▶); molecular graphics: *ORTEP-3* (Farrugia, 1997 ▶); software used to prepare material for publication: *PLATON* (Spek, 2009 ▶).

## Supplementary Material

Crystal structure: contains datablocks global, I. DOI: 10.1107/S1600536809047187/wn2366sup1.cif

Structure factors: contains datablocks I. DOI: 10.1107/S1600536809047187/wn2366Isup2.hkl

Additional supplementary materials:  crystallographic information; 3D view; checkCIF report

## Figures and Tables

**Table 1:** Hydrogen-bond geometry (Å, °)

*D*—H⋯*A*	*D*—H	H⋯*A*	*D*⋯*A*	*D*—H⋯*A*
N1—H1⋯O15^i^	0.83 (4)	2.20 (4)	2.957 (4)	152 (4)
N3—H3⋯S2^ii^	0.89 (5)	2.48 (5)	3.355 (3)	170 (4)
C46—H46⋯S2^iii^	0.95	2.84	3.761 (4)	165
C16—H16*A*⋯*Cg*1^iv^	0.98	2.86	3.660 (4)	139

Symmetry codes: (i) 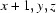; (ii) 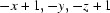; (iii) 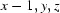; (iv) 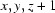. *Cg*1 is the centroid of the benzene ring.

**Fig. 1.**
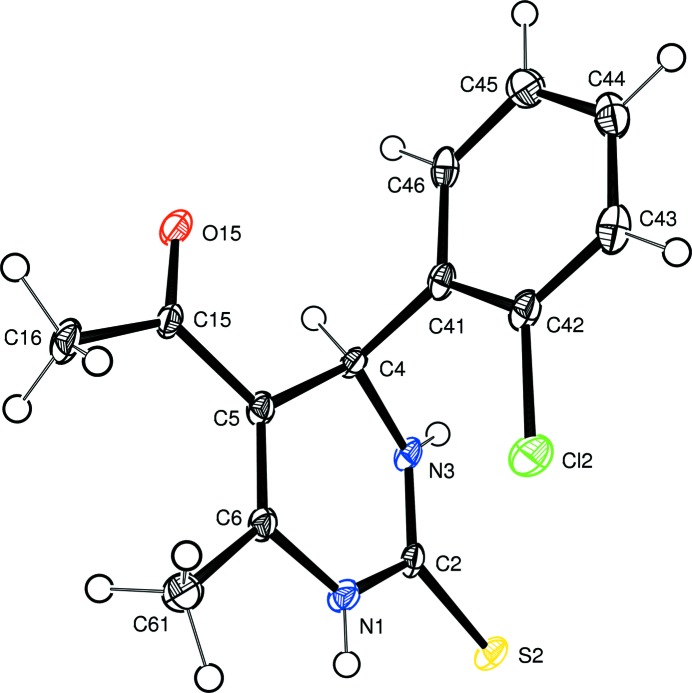
The molecular structure of the title compound, showing the atom-numbering scheme and displacement ellipsoids drawn at the 40% probability level. H atoms are shown as small spheres of arbitrary radius.

**Fig. 2.**
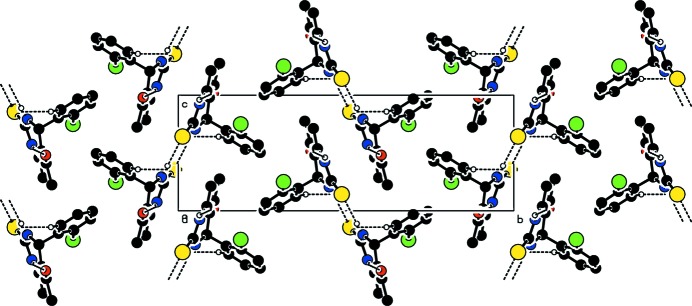
The packing of the title compound, viewed down the a axis. Dashed lines indicate hydrogen bonds. H atoms not involved in hydrogen bonding have been omitted.

## supplementary crystallographic information

### Comment

5-Ethoxycarbonyl-4-(3-hydroxyphenyl)-6-methyl-3,4-dihydropyrimidine- 2(1*H*)-thione can be used as an anticancer drug (Kappe *et al.*, 2000). Dihydropyrimidinones can be used as analgesic agents (Sadanandam *et al.*, 1992). Dihydropyrimidinones have attracted increasing attention due to their various therapeutic and pharmacological properties, such as antiviral, antibacterial, antihypertensive and antitumor effects (Kappe, 1993; Kappe, 2000). More recently they have emerged as integral backbones of several calcium blockers, antihypertensive agents, α-1a-antagonists and neuropeptide Y (NPY) antagonists (Atwal *et al.*, 1990; Rovnyak *et al.*, 1995). The crystal structures of three very closely related compounds have recently been reported [Anuradha *et al.*, (2008, 2009); Chitra *et al.*, (2009)]. This study of the title compound, was undertaken to compare the biological activity and structure of dihydropyrimidin-2(1*H*)-thione and its corresponding 2(1*H*)-one (Anuradha *et al.*, 2008).

In the title molecule, C_13_H_13_ClN_2_OS (Fig. 1) the heterocyclic ring adopts a flattened boat conformation with the plane through the four coplanar atoms (C2,N3,C5,C6) making a dihedral angle of 85.6 (1)° with the benzene ring, which adopts an axial orientation. The thionyl, acetyl and methyl groups all have equatorial orientations. Intermolecular N1—H1···O15(1 + *x*, *y*, *z*), N3—H3···S2(1 - *x*, -*y*, 1 - *z*) and C46—H46···S2(-1 + *x*, *y*, *z*) hydrogen bonds are found in the crystal structure. Furthermore, a weak C16—H16A···π(*x*, *y*, 1 + *z*) interaction involving the benzene ring (C41—C46) is also found.

### Experimental

A solution of acetylacetone (1.001 g, 0.01 mol), 2-chlorobenzaldehyde (1.406 g, 0.01 mol) and thiourea (1.149 g, 0.015 mol) was heated under reflux in the presence of calcium fluoride (0.078 g, 0.001 mol) for 1.5 h (monitored by TLC). After completion of the reaction, the reaction mixture was cooled to room temperature and poured into crushed ice. The crude product, containing also the catalyst, was collected on a Buchner funnel by filtration. The mixture of the product and the catalyst was digested in methanol (40 ml). The undissolved catalyst was removed by filtration. The crude product was obtained by evaporation of the methanol and further purified by recrystallization from hot ethanol to afford the pure title compound. Yield 84% (1.86 g).

### Refinement

H1 at N1 was located in a difference Fourier map and the N1—H1 distance was restrained to be 0.83 (4) Å. H3 at N3 was located in a difference Fourier map and refined freely; N3—H3 = 0.89 (5) Å. The remaining H atoms were positioned geometrically and allowed to ride on their parent atoms, with C—H = 0.95 - 1.00 Å; *U*_iso_(H) = k*U*_eq_(C), where k = 1.5 for methyl and 1.2 for all other H atoms. The maximum residual electron density peak is located 0.86 Å from C42.

### Crystal data

**Table:**

C_13_H_13_ClN_2_OS	*F*(000) = 584
*M**_r_* = 280.77	*D*_x_ = 1.468 Mg m^−^^3^
Monoclinic, *P*2_1_/*c*	Melting point: 484.5 K
Hall symbol: -P 2ybc	Cu *K*α radiation, λ = 1.54184 Å
*a* = 7.2346 (12) Å	Cell parameters from 3483 reflections
*b* = 22.585 (3) Å	θ = 5.7–73.8°
*c* = 8.0941 (15) Å	µ = 4.11 mm^−^^1^
β = 106.177 (17)°	*T* = 110 K
*V* = 1270.2 (4) Å^3^	Triangular-plate, colourless
*Z* = 4	0.45 × 0.43 × 0.12 mm

### Data collection

**Table:**

Oxford Diffraction Xcalibur Ruby Gemini diffractometer	2497 independent reflections
Radiation source: Enhance (Cu) X-ray Source	2246 reflections with *I* > 2σ(*I*)
graphite	*R*_int_ = 0.035
Detector resolution: 10.5081 pixels mm^-1^	θ_max_ = 74.1°, θ_min_ = 6.0°
ω scans	*h* = −8→8
Absorption correction: multi-scan (*CrysAlis PRO*; Oxford Diffraction, 2009)	*k* = −27→16
*T*_min_ = 0.451, *T*_max_ = 1.000	*l* = −9→8
4641 measured reflections	

### Refinement

**Table:**

Refinement on *F*^2^	Primary atom site location: structure-invariant direct methods
Least-squares matrix: full	Secondary atom site location: difference Fourier map
*R*[*F*^2^ > 2σ(*F*^2^)] = 0.062	Hydrogen site location: inferred from neighbouring sites
*wR*(*F*^2^) = 0.184	H atoms treated by a mixture of independent and constrained refinement
*S* = 1.13	*w* = 1/[σ^2^(*F*_o_^2^) + (0.1043*P*)^2^ + 2.5484*P*] where *P* = (*F*_o_^2^ + 2*F*_c_^2^)/3
2497 reflections	(Δ/σ)_max_ = 0.001
173 parameters	Δρ_max_ = 1.19 e Å^−^^3^
1 restraint	Δρ_min_ = −0.38 e Å^−^^3^

### Special details

**Table:**

Geometry. Bond distances, angles *etc*. have been calculated using the rounded fractional coordinates. All su's are estimated from the variances of the (full) variance-covariance matrix. The cell e.s.d.'s are taken into account in the estimation of distances, angles and torsion angles
Refinement. Refinement of *F*^2^ against ALL reflections. The weighted *R*-factor *wR* and goodness of fit *S* are based on *F*^2^, conventional *R*-factors *R* are based on *F*, with *F* set to zero for negative *F*^2^. The threshold expression of *F*^2^ > 2σ(*F*^2^) is used only for calculating *R*-factors(gt) *etc*. and is not relevant to the choice of reflections for refinement. *R*-factors based on *F*^2^ are statistically about twice as large as those based on *F*, and *R*- factors based on ALL data will be even larger.

### Fractional atomic coordinates and isotropic or equivalent isotropic displacement parameters (Å^2^)

**Table:**

	*x*	*y*	*z*	*U*_iso_*/*U*_eq_	
Cl2	0.66371 (12)	0.18610 (4)	0.75112 (12)	0.0277 (3)	
S2	0.80912 (11)	0.01280 (4)	0.64291 (10)	0.0198 (3)	
O15	0.1673 (3)	0.10482 (11)	1.0187 (3)	0.0216 (7)	
N1	0.7634 (4)	0.06358 (13)	0.9244 (3)	0.0185 (8)	
N3	0.4871 (4)	0.05026 (12)	0.7069 (3)	0.0162 (8)	
C2	0.6750 (5)	0.04420 (15)	0.7613 (4)	0.0164 (9)	
C4	0.3680 (4)	0.08463 (14)	0.7922 (4)	0.0150 (8)	
C5	0.4719 (5)	0.09279 (14)	0.9816 (4)	0.0156 (9)	
C6	0.6658 (5)	0.08508 (15)	1.0377 (4)	0.0168 (9)	
C15	0.3405 (5)	0.10641 (14)	1.0875 (4)	0.0164 (9)	
C16	0.4118 (5)	0.12310 (17)	1.2760 (4)	0.0228 (10)	
C41	0.2962 (5)	0.14262 (14)	0.6951 (4)	0.0170 (9)	
C42	0.4137 (5)	0.18851 (15)	0.6678 (4)	0.0200 (10)	
C43	0.3389 (6)	0.23843 (16)	0.5701 (5)	0.0246 (10)	
C44	0.1437 (6)	0.24424 (16)	0.5040 (5)	0.0257 (11)	
C45	0.0217 (5)	0.20019 (17)	0.5320 (5)	0.0247 (10)	
C46	0.0963 (5)	0.15024 (15)	0.6247 (4)	0.0207 (10)	
C61	0.7992 (5)	0.09730 (19)	1.2130 (4)	0.0269 (10)	
H1	0.882 (5)	0.0629 (17)	0.962 (5)	0.012 (9)*	
H3	0.422 (7)	0.032 (2)	0.611 (7)	0.029 (11)*	
H4	0.25121	0.06016	0.78665	0.0180*	
H16A	0.30167	0.13196	1.31997	0.0342*	
H16B	0.49480	0.15808	1.28872	0.0342*	
H16C	0.48517	0.09005	1.34108	0.0342*	
H43	0.42279	0.26815	0.54971	0.0295*	
H44	0.09168	0.27837	0.43890	0.0309*	
H45	−0.11364	0.20451	0.48713	0.0297*	
H46	0.01111	0.12031	0.64156	0.0249*	
H61A	0.78706	0.13886	1.24330	0.0406*	
H61B	0.93214	0.08937	1.21223	0.0406*	
H61C	0.76544	0.07166	1.29790	0.0406*	

### Atomic displacement parameters (Å^2^)

**Table:**

	*U*^11^	*U*^22^	*U*^33^	*U*^12^	*U*^13^	*U*^23^
Cl2	0.0192 (5)	0.0300 (5)	0.0347 (5)	−0.0063 (3)	0.0090 (4)	0.0031 (3)
S2	0.0184 (4)	0.0252 (5)	0.0188 (4)	−0.0016 (3)	0.0102 (3)	−0.0050 (3)
O15	0.0169 (13)	0.0327 (13)	0.0173 (12)	−0.0004 (9)	0.0084 (9)	0.0002 (9)
N1	0.0148 (14)	0.0282 (15)	0.0131 (13)	−0.0005 (11)	0.0051 (11)	−0.0036 (10)
N3	0.0190 (14)	0.0206 (13)	0.0112 (12)	−0.0020 (11)	0.0077 (10)	−0.0052 (10)
C2	0.0192 (16)	0.0209 (15)	0.0111 (14)	−0.0027 (12)	0.0074 (12)	0.0005 (11)
C4	0.0131 (15)	0.0205 (15)	0.0132 (14)	−0.0014 (12)	0.0066 (12)	−0.0016 (11)
C5	0.0178 (16)	0.0187 (15)	0.0111 (14)	−0.0008 (12)	0.0055 (12)	−0.0017 (11)
C6	0.0159 (16)	0.0241 (16)	0.0106 (14)	−0.0018 (12)	0.0039 (12)	−0.0006 (12)
C15	0.0187 (17)	0.0182 (15)	0.0150 (15)	−0.0010 (12)	0.0092 (13)	0.0025 (11)
C16	0.0264 (18)	0.0319 (18)	0.0132 (16)	−0.0021 (14)	0.0107 (13)	−0.0041 (13)
C41	0.0213 (16)	0.0211 (16)	0.0101 (14)	−0.0030 (12)	0.0069 (12)	−0.0036 (12)
C42	0.0221 (17)	0.0250 (17)	0.0152 (16)	−0.0026 (13)	0.0091 (13)	−0.0016 (12)
C43	0.035 (2)	0.0221 (17)	0.0201 (17)	−0.0056 (14)	0.0131 (15)	−0.0015 (13)
C44	0.034 (2)	0.0243 (17)	0.0194 (18)	0.0016 (15)	0.0087 (15)	0.0035 (13)
C45	0.0230 (18)	0.0310 (18)	0.0186 (17)	0.0003 (14)	0.0032 (13)	−0.0010 (14)
C46	0.0294 (19)	0.0229 (16)	0.0120 (15)	−0.0033 (13)	0.0092 (13)	−0.0019 (12)
C61	0.0188 (17)	0.045 (2)	0.0155 (17)	0.0003 (15)	0.0024 (13)	−0.0052 (14)

### Geometric parameters (Å, °)

**Table:**

Cl2—C42	1.746 (4)	C41—C42	1.397 (5)
S2—C2	1.697 (4)	C42—C43	1.397 (5)
O15—C15	1.222 (4)	C43—C44	1.370 (6)
N1—C2	1.369 (4)	C44—C45	1.390 (6)
N1—C6	1.392 (4)	C45—C46	1.379 (5)
N3—C2	1.314 (5)	C4—H4	1.0000
N3—C4	1.467 (4)	C16—H16A	0.9800
N1—H1	0.83 (4)	C16—H16B	0.9800
N3—H3	0.89 (5)	C16—H16C	0.9800
C4—C41	1.541 (4)	C43—H43	0.9500
C4—C5	1.519 (4)	C44—H44	0.9500
C5—C6	1.360 (5)	C45—H45	0.9500
C5—C15	1.479 (5)	C46—H46	0.9500
C6—C61	1.503 (5)	C61—H61A	0.9800
C15—C16	1.516 (4)	C61—H61B	0.9800
C41—C46	1.410 (5)	C61—H61C	0.9800
			
Cl2···N1	3.095 (3)	C6···H16C	3.0900
Cl2···N3	3.304 (3)	C15···H43^viii^	2.9300
Cl2···C2	3.206 (4)	C16···H61A	2.8200
Cl2···C5	3.360 (4)	C16···H61C	2.7700
Cl2···C6	3.251 (3)	C16···H43^viii^	3.0800
Cl2···C45^i^	3.538 (4)	C41···H16A^v^	3.0600
Cl2···C46^i^	3.644 (4)	C42···H16A^v^	2.9900
Cl2···H45^i^	3.0400	C43···H16B^x^	2.9600
Cl2···H44^ii^	3.1500	C45···H61A^xi^	2.8400
S2···C16^iii^	3.604 (4)	C46···H44^viii^	3.0200
S2···N3^iv^	3.355 (3)	C61···H16B	2.8000
S2···H61C^v^	3.0300	C61···H16C	2.7500
S2···H46^i^	2.8400	H1···O15^i^	2.20 (4)
S2···H3^iv^	2.48 (5)	H1···H61B	2.0400
S2···H16C^iii^	3.1800	H3···S2^iv^	2.48 (5)
S2···H61B^vi^	3.0000	H4···O15	2.3600
O15···N1^vii^	2.957 (4)	H4···H46	2.2600
O15···C41	3.134 (4)	H16A···C41^ix^	3.0600
O15···C46	3.252 (4)	H16A···C42^ix^	2.9900
O15···C44^viii^	3.414 (4)	H16B···C61	2.8000
O15···H4	2.3600	H16B···H61A	2.2900
O15···H61B^vii^	2.6400	H16B···C43^viii^	2.9600
O15···H1^vii^	2.20 (4)	H16B···H43^viii^	2.5000
O15···H44^viii^	2.7300	H16C···C6	3.0900
N1···Cl2	3.095 (3)	H16C···C61	2.7500
N1···O15^i^	2.957 (4)	H16C···H61C	2.1900
N3···Cl2	3.304 (3)	H16C···S2^iii^	3.1800
N3···S2^iv^	3.355 (3)	H43···C15^x^	2.9300
C2···Cl2	3.206 (4)	H43···C16^x^	3.0800
C5···Cl2	3.360 (4)	H43···H16B^x^	2.5000
C6···Cl2	3.251 (3)	H44···Cl2^xiii^	3.1500
C15···C43^viii^	3.507 (5)	H44···O15^x^	2.7300
C16···S2^iii^	3.604 (4)	H44···C46^x^	3.0200
C16···C43^viii^	3.514 (5)	H45···Cl2^vii^	3.0400
C16···C42^ix^	3.495 (5)	H45···H61A^xi^	2.4100
C16···C61	3.042 (5)	H46···S2^vii^	2.8400
C41···O15	3.134 (4)	H46···H4	2.2600
C42···C16^v^	3.495 (5)	H61A···C16	2.8200
C43···C15^x^	3.507 (5)	H61A···C45^xii^	2.8400
C43···C16^x^	3.514 (5)	H61A···H16B	2.2900
C44···O15^x^	3.414 (4)	H61A···H45^xii^	2.4100
C45···C61^xi^	3.513 (5)	H61B···O15^i^	2.6400
C45···Cl2^vii^	3.538 (4)	H61B···H1	2.0400
C46···Cl2^vii^	3.644 (4)	H61B···S2^vi^	3.0000
C46···O15	3.252 (4)	H61C···S2^ix^	3.0300
C61···C16	3.042 (5)	H61C···C16	2.7700
C61···C45^xii^	3.513 (5)	H61C···H16C	2.1900
			
C2—N1—C6	124.1 (3)	C42—C43—C44	119.5 (4)
C2—N3—C4	125.9 (3)	C43—C44—C45	120.0 (3)
C2—N1—H1	121 (3)	C44—C45—C46	120.3 (4)
C6—N1—H1	115 (3)	C41—C46—C45	121.5 (3)
C2—N3—H3	119 (3)	N3—C4—H4	107.00
C4—N3—H3	115 (3)	C5—C4—H4	107.00
S2—C2—N3	123.7 (2)	C41—C4—H4	107.00
N1—C2—N3	116.9 (3)	C15—C16—H16A	109.00
S2—C2—N1	119.4 (3)	C15—C16—H16B	109.00
N3—C4—C5	110.4 (3)	C15—C16—H16C	109.00
N3—C4—C41	111.6 (3)	H16A—C16—H16B	109.00
C5—C4—C41	114.4 (3)	H16A—C16—H16C	110.00
C4—C5—C15	113.1 (3)	H16B—C16—H16C	109.00
C6—C5—C15	127.1 (3)	C42—C43—H43	120.00
C4—C5—C6	119.7 (3)	C44—C43—H43	120.00
N1—C6—C5	119.4 (3)	C43—C44—H44	120.00
N1—C6—C61	112.1 (3)	C45—C44—H44	120.00
C5—C6—C61	128.6 (3)	C44—C45—H45	120.00
C5—C15—C16	122.8 (3)	C46—C45—H45	120.00
O15—C15—C5	118.2 (3)	C41—C46—H46	119.00
O15—C15—C16	119.0 (3)	C45—C46—H46	119.00
C4—C41—C46	118.2 (3)	C6—C61—H61A	109.00
C42—C41—C46	116.5 (3)	C6—C61—H61B	109.00
C4—C41—C42	125.3 (3)	C6—C61—H61C	109.00
Cl2—C42—C41	121.8 (3)	H61A—C61—H61B	109.00
Cl2—C42—C43	116.1 (3)	H61A—C61—H61C	109.00
C41—C42—C43	122.1 (3)	H61B—C61—H61C	109.00
			
C6—N1—C2—S2	−174.1 (3)	C15—C5—C6—N1	170.2 (3)
C6—N1—C2—N3	4.6 (5)	C15—C5—C6—C61	−10.3 (6)
C2—N1—C6—C5	−6.2 (5)	C4—C5—C15—O15	6.0 (4)
C2—N1—C6—C61	174.3 (3)	C4—C5—C15—C16	−173.0 (3)
C4—N3—C2—S2	−170.3 (2)	C6—C5—C15—O15	−171.3 (3)
C4—N3—C2—N1	11.1 (5)	C6—C5—C15—C16	9.7 (5)
C2—N3—C4—C5	−22.0 (4)	C4—C41—C42—Cl2	−2.6 (5)
C2—N3—C4—C41	106.5 (3)	C4—C41—C42—C43	176.3 (3)
N3—C4—C5—C6	18.9 (4)	C46—C41—C42—Cl2	178.6 (2)
N3—C4—C5—C15	−158.6 (3)	C46—C41—C42—C43	−2.5 (5)
C41—C4—C5—C6	−108.0 (4)	C4—C41—C46—C45	−178.1 (3)
C41—C4—C5—C15	74.5 (4)	C42—C41—C46—C45	0.8 (5)
N3—C4—C41—C42	−61.9 (4)	Cl2—C42—C43—C44	−178.4 (3)
N3—C4—C41—C46	117.0 (3)	C41—C42—C43—C44	2.7 (5)
C5—C4—C41—C42	64.4 (4)	C42—C43—C44—C45	−1.0 (6)
C5—C4—C41—C46	−116.8 (3)	C43—C44—C45—C46	−0.7 (6)
C4—C5—C6—N1	−6.9 (5)	C44—C45—C46—C41	0.8 (5)
C4—C5—C6—C61	172.5 (3)		

Symmetry codes: (i) *x*+1, *y*, *z*; (ii) *x*+1, −*y*+1/2, *z*+1/2; (iii) −*x*+1, −*y*, −*z*+2; (iv) −*x*+1, −*y*, −*z*+1; (v) *x*, *y*, *z*−1; (vi) −*x*+2, −*y*, −*z*+2; (vii) *x*−1, *y*, *z*; (viii) *x*, −*y*+1/2, *z*+1/2; (ix) *x*, *y*, *z*+1; (x) *x*, −*y*+1/2, *z*−1/2; (xi) *x*−1, *y*, *z*−1; (xii) *x*+1, *y*, *z*+1; (xiii) *x*−1, −*y*+1/2, *z*−1/2.

### Hydrogen-bond geometry (Å, °)

**Table:**

*D*—H···*A*	*D*—H	H···*A*	*D*···*A*	*D*—H···*A*
N1—H1···O15^i^	0.83 (4)	2.20 (4)	2.957 (4)	152 (4)
N3—H3···S2^iv^	0.89 (5)	2.48 (5)	3.355 (3)	170 (4)
C46—H46···S2^vii^	0.95	2.84	3.761 (4)	165
C16—H16A···Cg1^ix^	0.98	2.86	3.660 (4)	139

Symmetry codes: (i) *x*+1, *y*, *z*; (iv) −*x*+1, −*y*, −*z*+1; (vii) *x*−1, *y*, *z*; (ix) *x*, *y*, *z*+1.
